# Inflammasome-Mediated Neuroinflammation: A Key Driver in Alzheimer’s Disease Pathogenesis

**DOI:** 10.3390/biom15050676

**Published:** 2025-05-07

**Authors:** Julie McGroarty, Shelbi Salinas, Hayden Evans, Bryan Jimenez, Vincent Tran, Samuel Kadavakollu, Arti Vashist, Venkata Atluri

**Affiliations:** 1 Department of Biomedical Sciences, Noorda College of Osteopathic Medicine, 2162 S 180 E, Provo, UT 84606, USA; do27.jamcgroarty@noordacom.org (J.M.); do27.sjsalinas@noordacom.org (S.S.); do27.hcevans@noordacom.org (H.E.); do27.brjimenez@noordacom.org (B.J.); do27.vqtran@noordacom.org (V.T.); 2 The Office of Academic Affairs, Meritus School of Osteopathic Medicine, 11120 Health Drive, Hagerstown, MD 21742, USA; samuel.kadavakollu@msom.org; 3 Department of Cellular and Molecular Medicine, Herbert Wertheim College of Medicine, Florida International University, Miami, FL 33199, USA; avashist@fiu.edu; 4 Department of Biomedical Sciences, Meritus School of Osteopathic Medicine, 11120 Health Drive, Hagerstown, MD 21742, USA

**Keywords:** Alzheimer’s disease, neuroinflammation, NLRP3 inflammasome, amyloid beta, tau pathology, microglia activation, synaptic dysfunction, cytokines, neurodegeneration, therapeutic targets

## Abstract

Alzheimer’s disease (AD) is a progressive neurodegenerative disorder predominantly affecting the elderly, characterized by memory loss, cognitive decline, and functional impairment. While hallmark pathological features include extracellular amyloid beta (Aβ) plaques and intracellular neurofibrillary tangles composed of hyperphosphorylated tau protein, increasing evidence points to chronic neuroinflammation as a key driver of disease progression. Among inflammatory mechanisms, the activation of the NLRP3 (nucleotide-binding domain, leucine-rich repeat, and pyrin domain-containing protein 3) inflammasome in microglia plays a pivotal role by amplifying neuroinflammatory cascades, exacerbating synaptic dysfunction, and accelerating neuronal loss. This review examines the molecular underpinnings of AD with a focus on NLRP3 inflammasome-mediated neuroinflammation, detailing the crosstalk between Aβ, tau pathology, and innate immune responses. Finally, we highlight emerging therapeutic strategies targeting NLRP3 inflammasome activation as promising avenues for mitigating neuroinflammation and slowing AD progression.

## 1. Introduction

Alzheimer’s disease (AD) is one of the most prevalent neurodegenerative disorders, primarily affecting the elderly and manifesting as progressive cognitive impairment. Its clinical symptoms commonly include memory loss, confusion, mood disturbances, and difficulties in reasoning, ultimately resulting in a significant decline in an individual’s quality of life. Despite decades of intensive research, the exact pathophysiological mechanisms underlying AD remain incompletely understood. While various hypotheses have been proposed and debated, one consistent observation is the presence of key misfolded proteins in the brains of individuals with AD. These hallmark features include extracellular amyloid beta (Aβ) deposits and intracellular neurofibrillary tangles (NFTs), which arise from the aggregation of hyperphosphorylated tau protein [1]. The accumulation of these pathological proteins induces cellular stress, triggering a cascade of neuroinflammatory responses.

Among these responses, the production of proinflammatory cytokines, such as interleukin-1β (IL-1β) and interleukin-18 (IL-18), plays a central role. These cytokines are activated through the assembly of the inflammasome complex, a multimeric structure composed of pattern recognition receptors (PRRs), an adaptor protein, and the effector enzyme pro-caspase-1. In the brain, PRRs (the essential components of the innate immune system, responsible for recognizing pathogen-associated molecular patterns and damage-associated molecular patterns) are primarily expressed by microglia, macrophages, astrocytes, and, to a lesser extent, oligodendrocytes and are located either on the cell membrane (e.g., Toll-like receptors, TLRs) or within the cytoplasm (e.g., NOD-like receptors, NLRs) [2,3,4]. Upon activation, NLRs initiate the assembly of inflammasomes, which are cytosolic multiprotein complexes. These inflammasomes facilitate the activation of pro-inflammatory caspases, particularly caspase-1, leading to the maturation and secretion of key inflammatory cytokines, including interleukin (IL)-1β and IL-18 [5].

The activation of the nucleotide-binding oligomerization domain-like receptor pyrin domain-containing 3 (NLRP3) inflammasome is a tightly regulated, multistep process that occurs in a defined sequence. The first phase, known as priming, is followed by an activation step involving post-transcriptional and post-translational regulation. During priming, signaling through cytokine receptors or TLRs activates NF-κB, which translocates to the nucleus to drive the transcription and translation of NLRP3 and pro-IL-1β. At this stage, NLRP3 remains in an autoinhibited state. A second signal, such as ATP, potassium efflux, or lysosomal damage, is required to relieve this inhibition and promote the assembly of the NLRP3 inflammasome complex. This activation is facilitated by post-translational modifications of NLRP3, such as phosphorylation, and by its interaction with NEK7 (NIMA-related kinase 7), a critical regulator that binds NLRP3 to promote inflammasome assembly. These processes drive the conformational changes required for full inflammasome activation [6,7].

The NLRP3 inflammasome has been the most studied, and we heavily target its discussion in this review. Experimental models have demonstrated that various components of AD pathology, including Aβ plaques and tau tangles, can activate the NLRP3 inflammasome pathway. In this review, we explore the molecular mechanisms by which these pathological stressors trigger inflammasome activation, discuss their role in AD progression, and examine potential therapeutic strategies aimed at modulating inflammasome activity to slow or prevent disease advancement.

## 2. Molecular Mechanisms of Alzheimer’s Disease Development

Alzheimer’s disease (AD) is a progressive neurodegenerative disease. Significant histological characteristics of AD include neuroinflammation, extracellular aggregates of amyloid beta (Aβ) plaques, and intracellular neurofibrillary tangles (NFTs). The precise molecular mechanisms underlying AD’s development are still not fully understood. However, several key factors and hypotheses have been suggested. The inflammation hypothesis involves several diseases and lifestyles, such as obesity, diabetes, and hypertension, associated with inflammation and immunity being linked with risk factors for developing AD [8]. A predominant hypothesis, the amyloid-cascade hypothesis, emphasizes the accumulation of Aβ in the brain.

**Figure 1:**
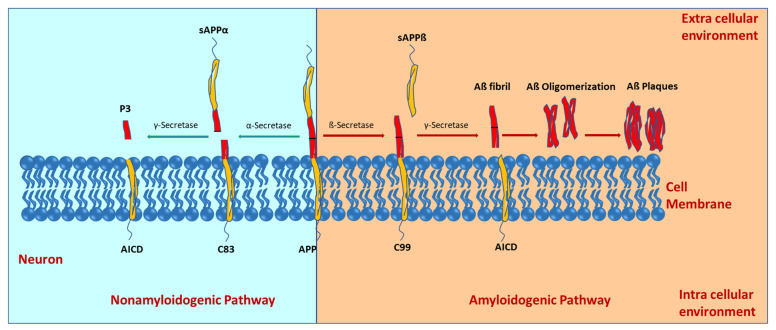
Alternative splicing and processing of Amyloid Precursor Protein (APP) through amyloidogenic and non-amyloidogenic pathways. In the non-amyloidogenic pathway, APP is sequentially cleaved by α-secretase and γ-secretase. Cleavage by α-secretase produces soluble APPα (sAPPα) and a membrane-bound C-terminal fragment (CTFα or C83). Subsequent cleavage of C83 by γ-secretase generates non-toxic P3 peptides and the APP intracellular domain (AICD). In contrast, the amyloidogenic pathway involves the initial cleavage of APP by β-secretase, releasing soluble APPβ (sAPPβ) and generating the membrane-associated CTF99/89 fragment. γ-secretase then cleaves CTF99/89 to produce various Amyloid Beta (Aβ) peptides, predominantly Aβ40 and Aβ42, with Aβ42 being more prone to aggregation. Aggregated Aβ42 forms insoluble amyloid fibrils, leading to plaque deposition. This process also triggers kinase activation, resulting in the hyperphosphorylation of tau proteins and the subsequent formation of neurofibrillary tangles (NFTs), contributing to Alzheimer’s disease pathology.

The amyloid precursor protein (APP) is a type I transmembrane highly conserved, single-pass transmembrane glycoprotein with large extracellular domains comprising 695–770 amino acids. The alternative splicing of APP transcripts produces various mRNA isoforms encoding proteins ranging from 695 to 770 amino acids in length. The three most common isoforms are APP695, APP751, and APP770, all of which contain the amyloid-beta (Aβ) region [9,10]. Under non-amyloidogenic processing, APP is sequentially cleaved by α-secretase and γ-secretase. The α-secretase can cleave APP at residue L688, yielding soluble α-APP (sAPPα) and the C-terminal fragment α (CTFα or C83). The subsequent γ-secretase cleavage of C83 generates non-toxic P3 peptides (which are soluble and has a role in normal synaptic signaling) and the APP intracellular domain (AICD) (Figure 1). γ-secretase is an aspartyl protease composed of presenilin 1 (PS1), anterior pharynx-defective 1, presenilin enhancer 2 (PEN-2), and nicastrin [11]. Aβ pathology emerges due to the incorrect cleavage of APP in amyloidogenic pathway by β- and γ-secretases. In this pathway, the APP is first cleaved by β-secretase, otherwise known as β-site cleavage enzyme 1 (BACE-1) producing soluble β-APP (sAPPβ) and the C-terminal fragment β (CTFβ or C99). The subsequent cleavage of C99 by γ-secretase generates the APP intracellular domain (AICD) and amyloid-β (Aβ) peptides [12,13] (Figure 1). Through cleavage, various lengths of Aβ peptides are generated. Two main types of Aβ peptides, Aβ1-40 and Aβ1-42, have been found to have a direct relationship with plaque formation and the induction of neurotoxicity. Aβ1-40 is relatively more abundant in the brain and less prone to aggregation than Aβ1-42 and is typically more soluble. Although less abundant, Aβ1-42 is highly insoluble, neurotoxic, and prone to aggregate into toxic Aβ oligomers [14]. It disrupts brain homeostasis, prompts neuroinflammation, advances brain degradation, and contributes to neuronal cell death.

Although it is not yet known whether it is a direct or indirect relationship, there is evidence that, in the disease state, Aβ1-42/Aβ1-40 aggregation leads to tau protein hyperphosphorylation, microtubule destabilization, and the formation of NFTs [1]. The normal function of tau includes stabilizing microtubules. However, the hyperphosphorylation of tau disrupts this normal function and destabilizes the neuronal cytoskeleton, impairing axonal transport and compromising neuronal function [15]. The loss of microtubule stability contributes to synaptic dysfunction and neuronal degeneration observed in AD. Hyperphosphorylated tau can also propagate from neuron to neuron [16]. Thus, over time, neurodegeneration can spread to different brain regions. Furthermore, NFTs are associated with neuronal dysfunction and cell death. Studies have shown that tau pathology has been implicated in impairing synaptic function [17]. Tau aggregates can interfere with synaptic transmission and plasticity, leading to AD-related cognitive deficits.

The aggregation of Aβ1-42/Aβ1-40 blocks ion channels and alters calcium homeostasis, elevating mitochondrial stress [18]. Consequently, the metabolism and regulation of glucose diminishes. Mitochondrial dysfunction and stress have been indicated to contribute to the pathogenesis of AD. Mitochondria are vital players in energy production and have significant roles in cellular metabolism, calcium homeostasis, and oxidative stress regulation. With the high energy demand of neurons, mitochondrial dysfunction can impair their ability to produce adenosine triphosphate (ATP), leading to energy deficits and impacting neuronal function and viability [19]. This energy deficit is particularly detrimental in brain regions crucial for memory and cognition, contributing to the cognitive decline observed in AD. In addition, mitochondria are a significant source of reactive oxygen species (ROS) production in cells. Oxidative stress and cellular damage can result from excessive ROS production by dysfunctional mitochondria. Oxidative stress can promote the aggregation of Aβ peptides, exacerbate tau pathology, and induce neuronal death [20,21].

Genetics also contribute to the development of AD. Early-onset familial AD (EOAD) represents less than 5% of AD cases and is characterized by the onset of symptoms before the age of 65 [22]. EOAD is typically associated with autosomal dominant inheritance and is often caused by mutations in the following three genes: amyloid precursor protein (APP), presenilin 1 (PSEN1), and presenilin 2 (PSEN2) [23]. Mutations in these genes lead to the overproduction or altered processing of Aβ peptides, contributing to amyloid plaque formation and neurodegeneration. Late-onset AD (LOAD), the most common form of AD, typically occurs after the age of 65 and involves multiple genetic and environmental factors [24]. The ε4 allele of the apolipoprotein E (APOE ε4) gene is the strongest genetic risk factor for LOAD. Carrying one copy of the APOE ε4 allele increases the risk of developing AD, while having two copies significantly increases the risk [25]. However, it is essential to note that not all individuals with the APOE ε4 allele develop AD, and many people with AD do not carry this allele.

In summary, AD is a multifactorial disorder characterized by multiple complex processes that contribute to its development and progression. Aβ peptide accumulation, tau pathology, oxidative stress, neuroinflammation, mitochondrial dysfunction, and genetic factors are thought to interact and exacerbate the neurodegeneration and cognitive decline seen in AD.

## 3. Systemic Inflammation and Microglial Dynamics in AD Progression

The interplay between systemic inflammation and microglial dynamics plays a crucial role in the progression of Alzheimer’s disease. This review integrates findings from several studies to elucidate how systemic inflammation influences microglial and other cells’ behavior in the brain, amyloid-beta (Aβ) pathology, and cognitive decline in AD.

In exploring the impact of systemic inflammation on microglial morphology and function, Tejera et al. (2019) [26] utilized Cx3cr1-eGFP and APP/PS1 mouse models with in vivo two-photon laser scanning microscopy (2PLSM) to reveal age-dependent microglial changes in Alzheimer’s disease (AD). Their findings demonstrated that older mice exhibited signs of microglial activation, such as reduced branching and increased soma volume, even prior to peripheral lipopolysaccharide (LPS) challenge. Systemic inflammation, induced by LPS, intensified Aβ deposition by impairing microglial phagocytosis and enhancing proliferation, shifts driven by the NLRP3 inflammasome. These morphological alterations coincided with a pro-inflammatory phenotype, marked by elevated cytokine production. Notably, microglia near Aβ deposits showed limited additional morphological changes following immune challenge, likely due to pre-existing activation, while distant microglia displayed dynamic responses, highlighting spatial heterogeneity in microglial behavior during systemic inflammation.

Further research has explored how secondary inflammatory responses in microglia, astrocytes, neurons, and human brain tissue contribute to Alzheimer’s disease (AD) progression. Lopez-Rodriguez et al. (2021) [27] utilized APP/PS1 double transgenic mouse models to demonstrate that microglia and astrocytes in the AD brain are primed, exhibiting exaggerated inflammatory responses to acute peripheral lipopolysaccharide (LPS) challenge. Specifically, microglia in APP/PS1 mice produced significantly elevated interleukin-1β (IL-1β) following systemic LPS administration, with plaque-associated microglia showing heightened IL-1β responses to both LPS and intracerebral IL-1β stimulation. Similarly, astrocytes displayed amplified chemokine (e.g., CCL2, CXCL1, CXCL10) and IL-6 responses to elevated IL-1β and peripheral LPS, suggesting an amplification loop that exacerbates neuroinflammation in the amyloid-laden brain. These hypersensitive astrocyte responses to systemic LPS indicate that such effects can be triggered externally, beyond the central nervous system. The study’s findings, corroborated by elevated IL-1β and IL-6 in AD patients with systemic infection, suggest that the AD brain is particularly vulnerable to intensified neuroinflammation during systemic inflammatory events, such as infection or injury, potentially accelerating disease progression.

Another area of interest in developing neuroinflammation in AD is the role of NLRP3-mediated microglial training. A study exploring NLRP3-mediated microglial training sheds light on its detrimental effects on Aβ clearance and cognitive decline in AD [28]. In this study, a sporadic Alzheimer’s disease (SAD) mouse model induced by streptozotocin (STZ) injection was utilized and demonstrated that NLRP3-mediated microglial training exacerbates microglial pro-inflammatory responses, while impairing Aβ phagocytosis. Conversely, the inhibition or deletion of microglial NLRP3 attenuates AD pathologies and mitigates the effects of microglial training, which offers potential therapeutic value for AD intervention.

A study investigating soluble Aβ species and their impact on NLRP3 inflammasome activation in microglia unveiled a crucial link between early Aβ aggregates and neuroinflammation [29]. Prior studies underscored fibrillar Aβ’s role in NLRP3 inflammasome activation [30], yet this study focuses on lower molecular aggregates preceding Aβ deposition. The data from this study showed that Aβ oligomers and protofibrils were phagocytosed by microglia with no difference in uptake from Aβ oligomers compared to protofibrils. Findings indicate that both Aβ oligomers and protofibrils induced a significant increase in IL-1β release, which indicates that they were able to activate the microglial NLRP3 inflammasome, resulting in the production of the pro-inflammatory cytokine, IL-1β [29]. This suggests an early neuroinflammatory response triggered by soluble Aβ species, implicating them in the initiation of AD pathogenesis.

Another protein that plays a critical role in the inflammatory response seen in Alzheimer’s disease is the transcription factor NF-κB [31,32]. In response to cellular stress, NF-κB regulates the DNA transcription of cytokines and other proteins. Lei et al. (2021) conducted a study to discover the role of NF-κB in the neuroinflammation seen in Alzheimer’s disease [33]. Serum samples of 112 patients with AD were compared to samples of 101 healthy control patients, and the protein levels of NF-κB, oxidative stress indices, and apoptosis regulators, as well as pyroptosis markers (NLRP3, caspase-1, and IL1β) were measured along with their mRNA expression levels [33]. In the patients with AD, it was found that levels of NF-κB and miR-146a-5p were highly expressed, while levels of apoptosis regulators were under-expressed. There was also a positive correlation between NF-κB and miR-146a-5p expression levels. At the same time, there was a negative correlation between NF-κB mRNA and apoptosis regulator mRNA levels in patients with AD [33]. The results of this study suggest that NF-κB upregulation promoted neuroinflammation in patients with Alzheimer’s disease. These diverse lines of inquiry highlight the intricate relationship between systemic inflammation, microglial dynamics, and AD pathology. By further understanding these complex interactions, researchers can develop novel therapeutic strategies to mitigate neuroinflammation and halt the progression of AD.

## 4. NLRP3 Inflammasome in AD Pathology

In Alzheimer’s disease, neuroinflammation is an immune response of the central nervous system mediated by microglia and astrocytes. While neuroinflammation is typically a protective response necessary for repairing brain tissue and preventing further damage, chronic inflammation can increase the risk of neurodegenerative disorders by perpetuating tissue injury and neural dysfunction [34,35].

**Figure 2:**
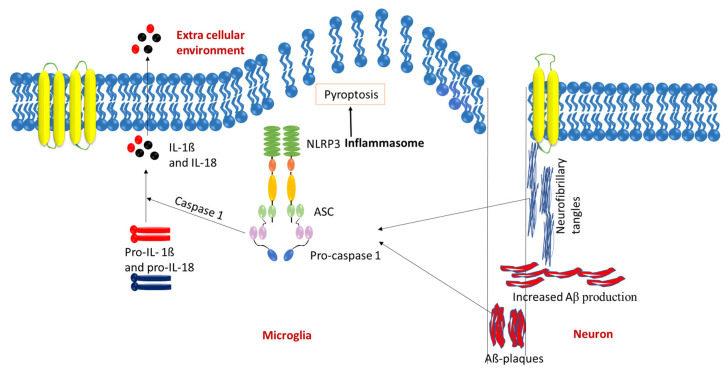
Activation of the inflammasome cascade by the deposition of Aβ plaques and neurofibrillary tangles. The accumulation of amyloid-beta (Aβ) plaques and neurofibrillary tangles in the brain triggers the recruitment of microglia around these pathological structures. This process initiates microglial activation and the subsequent stimulation of the NLRP3 inflammasome within these cells. Activated NLRP3 inflammasomes lead to the cleavage of pro-caspase-1 into its active form, caspase-1. Caspase-1 then processes pro-inflammatory cytokines pro-IL-1β and pro-IL-18 into their active forms. IL-1β serves as a potent pro-inflammatory mediator, while IL-18 promotes the production of interferon-gamma (IFN-γ), further amplifying the inflammatory response. These cytokines perpetuate neuroinflammation by activating additional microglia and astrocytes, creating a chronic inflammatory loop. Sustained inflammation ultimately contributes to synaptic dysfunction and neuronal degeneration, hallmark features of Alzheimer’s disease progression.

When amyloid-β deposits are engulfed by microglia, it initiates the NLRP3 inflammasome cascade that results in the release of the inflammatory mediator adaptor protein ASC (adapter protein apoptosis-associated speck-like protein containing a CARD) and cytokine IL-1β (Figure 2). When ASC is released, amyloid-β clusters around the adaptor protein to form composites that boost NLRP3 activity. This continued inflammation reduces microglia’s ability to clear amyloid plaques, resulting in pyroptotic cell death. During cell death, more ASC adaptor proteins are released, leading to a continued cycle of composite formations and NLRP3 activity, further impairing microglial cell ability for clearance and progressing AD [35,36,37]. In the context of AA (amyloid A) amyloidosis (due to disorders that create sustained inflammatory responses) [38], a condition marked by the deposition of serum amyloid A (SAA) that can be found in Alzheimer’s patients, ASC specks have been observed to co-localize with SAA in human cases and to enhance SAA fibril formation. A significant reduction in amyloid burden was observed in Pycard^−/−^ mice, which lack ASC, demonstrating its essential role in AD progression [39]. Additionally, Absence in Melanoma 2 (AIM2) is a crucial protein component of the inflammasome pathway. It interacts with ASC to trigger the activation of caspase-1, leading to the secretion of IL-1β. AIM2 expression is significantly elevated in the microglia of AD mouse models. The deletion of AIM2 in microglia has been demonstrated to improve cognitive impairment and synaptic deficits in an Aβ1-42-induced AD mouse model, suggesting the activation of microglia-mediated by AIM2 contributes to the inflammasome-mediated neuroinflammatory processes seen in AD [40].

After discovering that amyloid-β triggers NLRP3 signaling in vitro, Heneka et al. provided evidence suggesting that the NLRP3 inflammasome is crucial in driving Alzheimer’s disease in vivo [34]. Their research replicated the increased cleaved caspase-1 and IL-1β typically found in AD patient brains in APP/PS1 mice. These mice express a chimeric amyloid precursor protein and human presenilin-1, previously found to have mutations associated with the development of Alzheimer’s disease [34,36]. The APP/PS1 mice were crossed with mice deficient in NLRP3 gene expression to obtain APP/PS1/NLRP3^−/−^ mice. The APP/PS1 and APP/PS1/NLRP3^−/−^ mice completed water mazes and object recognition tests to assess spatial memory formation and deficits. The APP/PS1/NLRP3^−/−^ mice demonstrated no spatial memory impairment, while the APP/PS1 had severe deficits in spatial memory formation. The NLRP3^−/−^ mice demonstrated the prevention of the reduction in spine density, reduced hyperdynamic phenotype, normal habituation, improved neurobehavioral disturbances, and unaltered short-term plasticity and synaptic transmission. In contrast, the mice with NLRP3 expression exhibited spatial memory impairment, reduced spine density, increased locomotion, and slowed habituation. These observations indicate that NLRP3 inflammation has a significant impact on the cognitive and behavioral impairment seen in patients with Alzheimer’s disease [34].

Upon further analysis, it was discovered that mice lacking NLRP3 exhibited a significant reduction in Aβ deposition in the hippocampus and cortex. Impressively, the APP/PS1/NLRP3^−/−^ mice displayed a reduction of 70% in the highly aggregated, formic acid (FA) extractable forms of Aβ in the brain. Furthermore, there was a marked decrease in the size and volume of the outer regions of Aβ plaques, resulting in smaller plaque sizes on average. In contrast, microglial cells in APP/PS1 mice exhibited a lower degree of Aβ phagocytosis [34].

With this understanding of NLRP3-mediated inflammation in AD progression, recent research has explored the role of multiple factors that affect NLRP3, such as glia maturation factor (GMF). A study utilizing the immunofluorescence staining of post-mortem human brain tissue revealed the increased expression of NLRP3, caspase-1, and IL-18, all co-localizing with GMF and aggregated Aβ, as well as hyper-phosphorylated tau, suggesting impaired lysosomal clearance and autophagic dysfunction. Elevated levels of GMF and inflammasome components contribute to increased reactive oxygen species (ROS), triggering ROS-dependent NLRP3 inflammasome activation and lysosomal damage [41]. Additionally, tau proteins, in their phosphorylated forms, enhance NLRP3 acetylation, leading to inflammasome activation in microglial cells. While this mechanism is linked to cognitive deficits in animal models, the inhibition of the tau and NLRP3 interaction effectively reduces NLRP3 acetylation and cognitive impairment [42]. FUBP3 (Far Upstream Binding Protein 3) is another crucial transcription factor that enhances NLRP3 expression in neurons in the presence of Aβ and is also involved in tau phosphorylation. Also, elevated FUBP3 levels in the cortical neurons of aged wild-type mice and AD models suggest that it could serve as a potential therapeutic candidate for preventing the progression of AD [43].

The study by Liu et al. [44] adds to this understanding by highlighting the significant role of toll-like receptor 4 (TLR4) in activating the NLRP3 inflammasome in response to Aβ. Treating microglia with a TLR4 inhibitor significantly reduced Aβ1-42-induced NLRP3 activation, evidenced by decreased caspase-1 activity and IL-1β secretion, although NLRP3 activators could reverse this effect. Complementarily, research by Lučiūnaitė et al. shows that small Aβ protein clusters and early-stage fibrils can activate the NLRP3 inflammasome, leading to IL-1β release before the typical accumulation of Aβ plaques in AD [29].

Further exploring the NLRP3-mediated progression of AD, dynamin-related protein 1 (Drp1) has been identified as a disruptor of glycolytic homeostasis in oligodendrocytes (OLs), resulting in inflammatory damage and axonal damage. The inhibition of Drp1 not only corrects glycolytic deficits in mature OLs but also reduces NLRP3 inflammasome activation, alleviates myelin and axonal loss, and improves cognitive function in AD mouse models [45]. Recent findings also highlight the activation of the NLRP3 inflammasome in microglia and macrophages by the dipeptide glycine–arginine (GR), leading to IL-1β release and linking genetic factors associated with AD to neuroinflammation [46].

Apolipoprotein variants are another element that plays a role in neuroinflammation and neurodegeneration. Notably, carriers of the ApoE4 allele exhibit a pronounced pro-inflammatory response characterized by elevated levels of NLRP3 inflammasomes and ROS in microglia. This inflammatory effect is further exacerbated by combining ApoE4 with ATP, leading to a significant increase in pyroptosis compared to ApoE4 alone. The upregulation of NLRP3 inflammasomes in response to ApoE4 triggers NF-κB activation and contributes to mitochondrial autophagy dysfunction, which can be reversed through the knockout of NLRP3 [47]. Similarly, the activation of the NLRC4 inflammasome in microglia by ApoD has been shown to foster a pro-inflammatory effect. The inhibition of microglial ApoD can enhance the self-renewal capacity of neural stem cells (NSCs) and reduce neuronal apoptosis [48].

Caspases play an intricate role in modulating the NLRP3 inflammasome and its consequent impact on Alzheimer’s disease (AD) pathology. Caspase-8, for example, has been found to interact with the NLRP3 inflammasome and offers an alternative pathway for IL-1β activation. This pathway gains particular significance in scenarios where caspase-1, the traditional activator of IL-1β, is inhibited. Kumar et al. demonstrated that the absence of caspase-8 results in diminished Aβ deposition and microglial activation, possibly demonstrating its influence on the NLRP3 inflammasome in microglia and its production of IL-1β, contributing to AD’s neuroinflammatory aspects [49]. Additionally, caspase-4 has been shown to become overexpressed through the epigenetic mechanism of hypomethylation. The overexpression of caspase-4 is closely associated with activating various inflammatory components, including the NLRP3 inflammasome. The hypomethylation and subsequent overexpression of caspase-4 enhance the activation of the NLRP3 inflammasome, creating a feedback loop that drives neuroinflammation, as caspase-4 is predominantly expressed in microglia [50]. The role of microglial inflammation in worsening cognitive impairments in AD has also been highlighted through caspase-1′s functions. The genetic ablation of Casp1 or the inhibition by VX-765, a Casp1 inhibitor, demonstrates significant therapeutic potential. Specifically, VX-765 treatment initiated at the onset of cognitive deficits in AD mice models has shown an improvement in spatial memory impairments. This improvement is observed after the onset of cognitive deficits in aged mice with significant Aβ accumulation and microglial inflammation, showcasing the potential therapeutic benefits of targeting caspase activity in AD [51].

## 5. Therapeutic Molecules Targeting Inflammasomes in Alzheimer’s Disease

Pathologically, AD is characterized by the accumulation of Aβ plaques, neurofibrillary tangles, and chronic neuroinflammation. The NLRP3 inflammasome primarily mediates neuroinflammation, a critical component of the innate immune system in the inflammatory response [2]. Targeting the NLRP3 inflammasome presents a promising therapeutic strategy to mitigate AD pathology. This review synthesizes recent research on therapeutic molecules that inhibit NLRP3 inflammasome activation and their potential in treating AD. Table 1 compares various NLRP3 inflammasome inhibitors investigated for their potential in treating Alzheimer’s disease.

Among the various therapeutic agents targeting NLRP3 inflammasome, thioredoxin-1 is a naturally occurring antioxidant protein that mitigates neuroinflammation. It inhibits NLRP1-mediated pyroptosis, which is closely linked to NLRP3 inflammasome activity, thereby reducing neuronal death and neuroinflammation in AD models [52]. Natural compounds have gained significant attention for their capacity to inhibit NLRP3. The biflavonoid methylchamaejasmin, derived from *Khaya grandifoliola*, demonstrates anti-inflammatory effects by suppressing NLRP3 activation in the cell models of neuroinflammation [53]. Another particularly interesting molecule is colchicine, known for its use in treating gout. Recent studies on colchicine hybrids, such as SBN-284, have shown its dual action in inhibiting cholinesterase activity and suppressing NLRP3 inflammasome activation, making it a promising candidate for addressing both cholinergic dysfunction and neuroinflammation in AD [54]. Similarly, Gardenia jasminoides J. Ellis extract has been shown to attenuate memory impairment in Alzheimer’s disease models by inhibiting NLRP3 activation [55], showcasing the therapeutic potential of herbal remedies in combatting neurodegenerative conditions. Gastrodin, extracted from *Gastrodia elata*, has been shown to suppress NLRP3 inflammasome activation in neuroinflammation models, providing neuroprotection and alleviating neuropathic pain [56].

Salvianolic acid B, derived from *Salvia miltiorrhiza*, exhibits anti-inflammatory properties by modulating macrophage polarization, switching macrophages from the M1 (pro-inflammatory) phenotype to the M2 (anti-inflammatory) phenotype, effectively reducing NLRP3 inflammasome activation and illustrating the potential of natural compounds in combating AD [57]. Vitenegu acid, a compound isolated from the traditional Chinese medicinal plant V. negundo L., specifically targets NLRP3 without affecting other inflammasomes, inhibiting NLRP3 oligomerization, suppressing inflammasome activation, and demonstrating significant potential in both in vitro and in vivo models [58]. Epigallocatechin-3-gallate (EGCG), a polyphenol from green tea, inhibits NLRP3 activation through the ROS/TXNIP/NLRP3 pathway. By reducing oxidative stress and preventing inflammasome activation, EGCG has demonstrated neuroprotective effects in the models of Alzheimer’s disease [59]. This reinforces the connection between oxidative stress and neuroinflammation in AD.

Acacetin, a flavonoid found in plants like *Agastache rugosa,* blocks NLRP3 activation via the MAPK/NF-κB pathway, reducing oxidative stress and inflammation. This dual action underscores its therapeutic potential in neurodegenerative diseases like Alzheimer’s [60]. Echinatin, another natural flavonoid, inhibits NLRP3 inflammasome activation, reducing neuroinflammation and protecting neuronal health [61]. This adds to the growing list of flavonoids that may serve as effective therapeutic agents in AD.

Ghrelin, a hormone associated with hunger regulation, modulates autophagy to inhibit NLRP3 inflammasome activation, improving cognitive function in AD models [62]. This suggests that targeting metabolic pathways may provide new avenues for reducing neuroinflammation in AD. Additionally, beta-hydroxybutyrate (BHB), a ketone body produced during fasting or ketogenic diets, reduces AD pathology through NLRP3 inhibition, suggesting a protective effect against neuroinflammation [63,64]. Further, 2-[2-(benzo[d]thiazol-2-yl)phenylamino]benzoic acid (BPBA) a novel synthetic hybrid, targets both Aβ aggregation and NLRP3 inflammasome activation simultaneously. BPBA is designed by integrating benzothiazole, which targets Aβ aggregation, with o-aminobenzoic acid, an NLRP3 inflammasome inhibitor analog. In vitro studies have demonstrated BPBA’s efficacy in inhibiting Aβ aggregation and reducing inflammatory cytokine IL-1β levels. Animal studies further support BPBA’s potential, as it decreased Aβ oligomer levels and improved cognitive function in AD mouse models [65].

Proprotein convertase subtilisin/kexin type 9 (PCSK9), traditionally associated with cholesterol metabolism, has emerged as a novel target for reducing NLRP3 inflammasome activation by decreasing microglial activation and NLRP3 expression. Knockout studies in AD mouse models have shown that the absence of PCSK9 reduced Aβ deposition and neuroinflammation, suggesting its potential as a dual therapeutic target for managing metabolic and inflammatory pathways in AD [66]. NT-0796, a brain-penetrant small molecule, blocks the oligomerization of NLRP3, preventing the assembly of the inflammasome complex and thereby reducing the release of inflammatory cytokines. Preclinical studies have shown its potential in mitigating neuroinflammatory disorders, particularly AD [67].

Sulfonylureas, including glibenclamide, traditionally used for managing type 2 diabetes, also inhibit the NLRP3 inflammasome through interactions with ATP-sensitive potassium channels (K_ATP channels), reducing neuroinflammation in preclinical models [61]. Recent advancements in drug delivery, such as glibenclamide-loaded engineered nanovectors (GNVs), offer a sophisticated method for delivering anti-inflammatory drugs to the brain. These nanovectors enhance the efficacy of glibenclamide by promoting autophagy and reducing NLRP3 activation, illustrating the potential of nanotechnology in treating complex neurodegenerative disorders like Alzheimer’s disease [68].

Nicotinamide mononucleotide (NMN), an NAD+ precursor, represents a promising therapeutic strategy by enhancing mitochondrial function, reducing oxidative stress, and modulating NLRP3 inflammasome activation, with preclinical AD models showing improved cognitive function and reduced neuroinflammation [69]. Additionally, NLRP3 inflammasome inhibitors like Tranilast [70,71] and Oridonin [72,73] have demonstrated potential in preclinical studies for alleviating neuroinflammation and cognitive deficits in Alzheimer’s disease (AD) models. In addition to direct NLRP3 inhibitors, several therapeutic agents target downstream pathways of the NLRP3 inflammasome, focusing on mitigating oxidative stress and improving cellular health. Resveratrol, a polyphenol found in grapes, modulates neuroinflammatory pathways, including NLRP3, via SIRT1 activation and antioxidative effects, making it a promising candidate for AD therapy [74].

**Table 1:** NLRP3 inflammasome inhibitors targeting Alzheimer’s disease.

Inhibitor	Class	Mechanism of Action	AD-Specific Effects/Findings
Thioredoxin-1 [52]	Antioxidant protein	Inhibits NLRP1-mediated pyroptosis, linked to NLRP3 activity	Reduces neuronal death and neuroinflammation in AD models
Methylchamaejasmin [53]	Biflavonoid (natural)	Suppresses NLRP3 activation, reducing inflammatory response	Anti-inflammatory effects in cell models of neuroinflammation relevant to AD
Colchicine (SBN-284) [54]	Colchicine hybrid	Inhibits cholinesterase and suppresses NLRP3 activation	Promising candidate for addressing cholinergic dysfunction and neuroinflammation in AD
Acacetin [60]	Flavonoid (natural)	Blocks NLRP3 via MAPK/NF-κB pathway, reduces oxidative stress	Decreases inflammation in AD-relevant models
Echinatin [61]	Flavonoid (natural)	Inhibits NLRP3 activation, reducing neuroinflammation	Protects neuronal health in AD models
Ghrelin [62]	Hormone	Upregulates autophagy, inhibits NLRP3 activation	Improves cognitive function in AD models by reducing inflammation
Beta-hydroxybutyrate (BHB) [63,64]	Ketone body	Reduces NLRP3 activity, modulates neuroinflammation	Neuroprotective in AD models, influences metabolic pathways
Gardenia jasminoides extract [55]	Natural compound	Inhibits NLRP3 activation	Attenuates memory impairment in AD models
Gastrodin [56]	Natural compound	Suppresses NLRP3 activation	Provides neuroprotection and alleviates neuropathic pain in AD models
Oridonin [72,73]	Natural compound (diterpenoid)	Covalently inhibits NLRP3, blocking NEK7 interaction and inflammasome activation	Reduces synaptic loss, neuroinflammation, and cognitive deficits in AD models
Salvianolic acid B [57]	Natural compound	Modulates macrophage polarization (M1 to M2), reduces NLRP3 activation	Anti-inflammatory effects in AD models
Vitenegu acid [58]	Natural compound	Specifically inhibits NLRP3 oligomerization	Potential candidate to suppress inflammasome activation in AD
Epigallocatechin-3-gallate (EGCG) [59]	Polyphenol (natural)	Inhibits NLRP3 via ROS/TXNIP/NLRP3 pathway, reduces oxidative stress	Neuroprotective effects in AD models by reducing inflammation and oxidative stress
Resveratrol [74]	Polyphenol (natural)	Modulates NLRP3 and neuroinflammatory pathways, antioxidative	Reduces inflammation and activates protective mechanisms in AD models
PCSK9 [66]	Protein target	Reduces NLRP3 activation when knocked out	Decreases Aβ deposition and neuroinflammation in AD mouse models
NT-0796 [67]	Small molecule	Blocks NLRP3 oligomerization, preventing inflammasome assembly and cytokine release	Reduces neuroinflammation in preclinical AD models, brain-penetrant with therapeutic promise
Tranilast [70,71]	Small molecule	Inhibits NLRP3 by binding the NACHT domain, reducing inflammasome activation	Alleviates neuroinflammation and cognitive deficits in AD mouse models
Glibenclamide [61]	Sulfonylurea	Inhibits NLRP3 activation via ATP-sensitive potassium channels (K_ATP)	Inhibits NLRP3 and promotes autophagy in AD
BPBA [65]	Synthetic hybrid	Inhibits Aβ aggregation and NLRP3 activation (benzothiazole + o-aminobenzoic acid)	Reduces Aβ oligomers and IL-1β levels and improves cognition in AD mouse models
Nicotinamide mononucleotide (NMN) [69]	Synbiotic	Enhances mitochondrial function, reduces NLRP3 activation	Improves cognition and reduces neuroinflammation in preclinical AD models
Extracellular vesicles (EVs) [75]	Nanotechnology (hiPSC-derived)	Modulates microglial activation, reduces NLRP3 activity	Preserves cognitive function by altering microglial inflammatory profile in AD models
Adiponectin (gene therapy) [76]	Gene therapy	Increases adiponectin expression, reduces NLRP3 activation	Improves cognitive outcomes and reduces neuroinflammation in AD models

In addition to conventional small molecules and natural compounds, cutting-edge therapeutic strategies have emerged, particularly in gene therapy and nanotechnology. Adiponectin gene therapy offers an innovative approach to reducing NLRP3 activation. By increasing adiponectin expression in the liver, this therapy reduces neuroinflammation in the brain and improves cognitive outcomes in AD models. The ability of adiponectin to regulate both metabolic and inflammatory processes underscores its potential in AD treatment [76].

Another groundbreaking approach involves extracellular vesicles (EVs) derived from human-induced pluripotent stem cells (hiPSC). These vesicles can modulate microglial activation, reduce NLRP3 activity, and preserve cognitive function in Alzheimer’s models. By altering the inflammatory profile of microglia, EVs provide a highly targeted and innovative therapy for neuroinflammatory conditions [75]. Continued research into these therapeutics will enhance our understanding of neuroinflammation in AD and lead to improved outcomes for patients suffering from this condition.

## 6. Inflammasome Inhibitors in Clinical Trials

Several therapeutic agents targeting the NLRP3 inflammasome, either directly or indirectly, have shown promise in preclinical AD models but have not yet advanced beyond early-phase clinical trials for AD, often due to challenges in brain penetration and trial design for neurodegenerative diseases. Table 2 lists important inflammasome inhibitors in clinical trials as potential therapeutics for Alzheimer’s disease (AD). Dapansutrile (OLT1177), a selective NLRP3 inhibitor has completed a Phase II trial for gout (NCT02104050) [77]. In APP/PS1 AD mouse models, Dapansutrile reduced neuroinflammation by decreasing IL-1β and microglial activation, leading to a lower Aβ plaque burden [78]. However, as of now, no human trials for AD have been initiated.

VX-765 (Belnacasan), a caspase-1 inhibitor capable of crossing the blood–brain barrier (BBB), blocks NLRP3-mediated IL-1β and IL-18 activation and has completed Phase II trials for epilepsy with mixed efficacy (NCT01048255) [79,80]. Preclinical studies in AD mouse models demonstrate that VX-765 reduces IL-1β and Aβ deposition and improves cognition, positioning it as a potential candidate for future AD trials [81]. Anakinra, an IL-1β receptor antagonist, has been tested in various inflammatory conditions [82], and although preclinical studies in APP/PS1 AD mouse models show it reduces IL-1β signaling [83], specific clinical trials targeting mild cognitive impairment or AD are limited, necessitating further research to determine its efficacy in this context.

Canakinumab, an anti-IL-1β monoclonal antibody, completed a Phase II clinical trial (NCT04795466) to evaluate its efficacy and safety for cognition in participants with mild cognitive impairment or mild AD [84]. Lenalidomide, a thalidomide derivative reduces proinflammatory cytokines, such as TNF-α, IL-1, IL-6, and IL-12 [85], and is being investigated in the MCLENA-1 Phase II trial to assess its efficacy in reducing neuroinflammation and cognitive decline in individuals with amnestic mild cognitive impairment (aMCI) due to AD (NCT04032626) [86]. Sargramostim, a recombinant human granulocyte-macrophage colony-stimulating factor (GM-CSF), reduces proinflammatory cytokines and enhances microglial Aβ phagocytosis. It has completed a Phase II trial (NCT01409915) showing safety and preliminary efficacy in patients with mild-to-moderate AD, with an ongoing Phase II trial (NCT04902703) further evaluating its effects [87].

**Table 2:** Inflammasome inhibitors in clinical trials.

Inhibitor	Class	Mechanism of Action	AD-Specific Effects/Findings
Canakinumab [84]	Monoclonal antibody	Neutralizes IL-1β, a key NLRP3 inflammasome product, reducing neuroinflammation	Completed Phase II trial (NCT04795466) for cognition in MCI/mild AD [84]
Anakinra [82,83]	Recombinant protein	Blocks IL-1β receptor, inhibiting NLRP3-driven inflammatory signaling	Preclinical reduction in IL-1β signaling in APP/PS1 AD models; no active AD trials, tested in other conditions [82,83]
Sargramostim [87]	Recombinant protein	Stimulates GM-CSF, suppresses proinflammatory cytokines, enhances microglial Aβ phagocytosis	Completed Phase II trial (NCT01409915), ongoing Phase II (NCT04902703) for mild/moderate AD; reduces inflammation [87]
Dapansutrile [77,78] (OLT1177)	Small molecule	Selectively inhibits NLRP3, reducing inflammasome activation and cytokine release	Completed Phase II trial for gout (NCT02104050); reduces IL-1β, microglial activation, and Aβ plaques in APP/PS1 AD models; no AD trials [77,78]
VX-765 (Belnacasan) [79,80,81,82]	Small molecule	Inhibits caspase-1, blocking NLRP3-mediated IL-1β and IL-18 activation, BBB-penetrant	Completed Phase II trial for epilepsy (e.g., NCT01048255); reduces IL-1β, Aβ deposition, and improves cognition in AD models; no AD trials [79,80,81]
Lenalidomide [85,86]	Thalidomide derivative	Reduces proinflammatory cytokines (TNF-α, IL-1β, IL-6, IL-12) linked to inflammasomes	Ongoing MCLENA-1 Phase II trial (NCT04032626) for aMCI due to AD; aims to reduce neuroinflammation and cognitive decline [85,86]

## 7. Conclusions

Alzheimer’s disease (AD) is a multifaceted neurodegenerative disorder driven by amyloid-β (Aβ) plaques, tau neurofibrillary tangles, and chronic neuroinflammation, with the NLRP3 inflammasome playing a central role in disease progression. This review highlights how Aβ aggregates and hyperphosphorylated tau trigger NLRP3 activation in microglia, releasing pro-inflammatory cytokines like IL-1β and IL-18 that exacerbate synaptic dysfunction, impair Aβ clearance, and accelerate neuronal loss. The resulting neuroinflammatory cascade, amplified by systemic inflammation and genetic factors, such as APOE ε4, establishes a self-perpetuating cycle that links proteinopathy to immune dysregulation, oxidative stress, and mitochondrial dysfunction, underscoring the complexity of AD pathogenesis.

Therapeutic targeting of the NLRP3 inflammasome offers a promising strategy to mitigate AD pathology. Small molecule inhibitors (e.g., NT-0796), natural compounds (e.g., epigallocatechin-3-gallate), and advanced approaches like gene therapy and nanotechnology demonstrate potential in reducing inflammation, enhancing neuronal health, and slowing cognitive decline. By disrupting this inflammatory cycle, these interventions address both the upstream triggers and downstream consequences of AD, paving the way for innovative treatments. Continued research into these mechanisms and therapeutic modalities is essential to transform AD management and improve patient outcomes.

## Data Availability

Not applicable.
